# Adverse events in different administration routes of semaglutide: a pharmacovigilance study based on the FDA adverse event reporting system

**DOI:** 10.3389/fphar.2024.1414268

**Published:** 2024-06-03

**Authors:** Kaibin Niu, Maoxia Fan, Wulin Gao, Chen Chen, Guohua Dai

**Affiliations:** ^1^ First Clinical Medical College, Shandong University of Traditional Chinese Medicine, Jinan, Shandong, China; ^2^ Department of Geriatric Medicine, Affiliated Hospital of Shandong University of Traditional Chinese Medicine, Jinan, Shandong, China

**Keywords:** semaglutide, subcutaneous, oral, adverse events, FDA

## Abstract

**Background:**

With the continuously increasing incidence of type 2 diabetes, glucagon-like peptide-1 (GLP-1) receptor agonists, known for their dual benefits of effectively controlling blood glucose levels while also reducing weight and lowering cardiovascular disease risks, have been widely employed in the treatment of this condition. In recent years, semaglutide has garnered significant attention as the only injectable and orally administered glucagon-like peptide-1 receptor agonist (GLP-1RA). However, it is important to note that different routes of administration may lead to varying adverse events in patients. The aim of this study is to compare the adverse event profiles of semaglutide across different routes of administration by analyzing the adverse event reporting system of the U.S. Food and Drug Administration (FDA). The findings from this analysis will provide valuable insights for clinical practice and drug surveillance.

**Methods:**

Data was extracted from the U.S. Food and Drug Administration Adverse Event Reporting System (FAERS) database, specifically focusing on the period from the fourth quarter of 2017 to the fourth quarter of 2023. A comparative analysis was conducted using disproportionality analysis, reporting odds ratio (ROR), and stratified analysis methods to assess and compare the signals of adverse events (AE) and the time to onset of adverse reactions associated with different routes of administration of semaglutide from 2017 to 2023.

**Results:**

A total of 22,287 adverse reaction records related to semaglutide were identified in the FAERS database. A comparative analysis was performed on 16,346 records of subcutaneous administration and 2,496 records of oral administration. Different routes of administration can lead to varying adverse reaction outcomes. Compared to oral administration, subcutaneous injection is more likely to result in adverse events related to the endocrine system. Oral administration is more likely to induce adverse events in the gastrointestinal system. Additionally, it significantly accelerates the onset of adverse reactions. The comparative analysis of all relevant results indicates that semaglutide can lead to different adverse reaction events depending on the route of administration. Furthermore, there are significant differences in the time of onset for these adverse reactions.

**Conclusion:**

Semaglutide exhibits variations in adverse reaction events and the time of onset across different routes of administration. Therefore, when selecting the route of administration for semaglutide, clinicians should consider the risk of adverse events and weigh them against the clinical benefits. Based on these considerations, appropriate guidance and recommendations can be provided to patients.

## Introduction

Type 2 diabetes is a prevalent chronic metabolic disorder characterized by insulin resistance and insufficient insulin secretion, leading to elevated blood glucose levels and an increased risk of various complications (Drucker et al., 2017; Drucker, 2018). GLP-1 receptor agonists, as a novel class of drugs, mimic the action of naturally occurring GLP-1 hormone. They induce glucose-mediated insulin secretion, reduce glucagon release, decrease hepatic glucose output, delay gastric emptying, increase satiety, and improve cardiovascular risk factors. Consequently, they have emerged as an important therapeutic option in the management of type 2 diabetes (Meier, 2012; DeFronzo et al., 2014; Nauck and Meier, 2019a; Romera et al., 2019). Semaglutide, being the only injectable and orally administered glucagon-like peptide-1 receptor agonist (GLP-1RA), has garnered significant attention (Nauck and Meier, 2019b). In December 2017, the injectable formulation of semaglutide received approval from the U.S. Food and Drug Administration (FDA) for blood glucose control in adults with type 2 diabetes. Subsequently, in September 2019, the oral tablet formulation of semaglutide also received approval from the U.S. Food and Drug Administration (FDA) for improving blood glucose control in patients with type 2 diabetes, in conjunction with diet and exercise. This made semaglutide the first oral GLP-1 medication to receive approval globally (Meier, 2021a). Subsequently, in January 2020, the FDA expanded the indications for semaglutide based on the results of the SUSTAIN-6 clinical trial. This trial demonstrated a statistically significant reduction in cardiovascular event risk in the semaglutide group compared to the placebo group (6.6% vs. 8.9%, hazard ratio 0.74; 95% confidence interval [CI], 0.58–0.95; *p* < 0.001) (Marso et al., 2016; Accessdata, 2017). Due to its long-acting duration, high selectivity, and good tolerability, semaglutide has found widespread use in clinical practice.

In truth, while semaglutide has shown favorable efficacy in improving blood glucose control and reducing cardiovascular risk, it is important not to overlook the associated adverse events (Smits and Van Raalte, 2021). In clinical practice, semaglutide can be administered via subcutaneous injection or oral route. Although both routes of administration are considered effective and safe, they may have an impact on the incidence and types of adverse events experienced by patients (Accessdata, 2017). Gaining a comprehensive understanding of the adverse event profiles associated with various routes of semaglutide administration is essential for informing clinical practice and safeguarding patient safety. With the widespread clinical application of semaglutide, it is important for healthcare professionals to pay attention to the potential adverse events associated with different routes of administration (Meier, 2021b; Gallwitz and Giorgino, 2021). This study, based on the FAERS (FDA Adverse Event Reporting System) database, utilized a disproportionality analysis and stratified analysis to comprehensively investigate the potential adverse events and their time of onset associated with semaglutide in different routes of administration (Montastruc et al., 2011). For clinicians, understanding the adverse event profiles associated with different routes of administration of semaglutide can provide valuable guidance when selecting the appropriate mode of drug administration based on individual patient characteristics and conditions. This consideration can help optimize treatment decisions and enhance patient safety.

## Materials and methods

### Data sources

This study is based on the FAERS database, and employs a methodological approach combining discriminant analysis and stratified analysis to comparatively investigate the adverse events (AEs) of semaglutide across different administration routes. The FAERS database, being the largest global system for adverse event data reporting, offers valuable information regarding the safety profile of specific drugs in real-world settings through data mining and analysis (Min et al., 2018; Shu et al., 2022a). Based on the approval date of semaglutide by the FDA, our study data will encompass all relevant data from the fourth quarter of 2017 to the fourth quarter of 2023.

**FIGURE 1:**
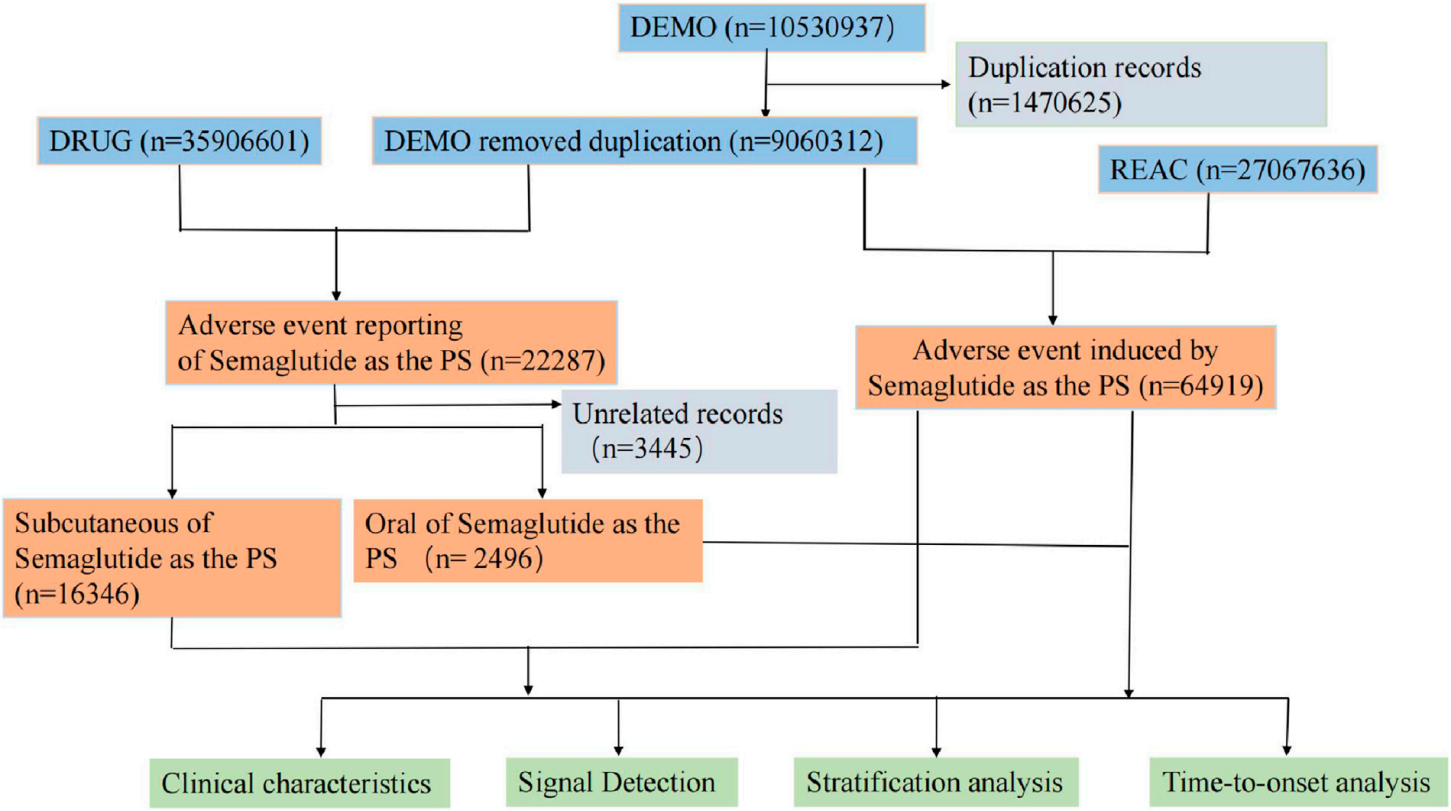
The process of screening semaglutide from the Food and Drug Administration adverse event reporting database for adverse events associated with different routes of administration.

During the data analysis phase, after excluding duplicate events, a total of 9,060,312 reports of adverse reaction events were obtained from the FAERS database. Among them, there were 22,287 cases related to semaglutide. Further screening was conducted to exclude cases with missing records and administration routes that were not relevant to the study. Ultimately, the study obtained a total of 16,346 adverse reaction events associated with subcutaneous injection and 2,496 events associated with oral administration, which were required for this research. The detailed flowchart outlining the multi-step process of data extraction, processing, and analysis is depicted in Figure 1.

## Results

### Descriptive analysis

In the FAERS database, from the fourth quarter of 2017 to the fourth quarter of 2023, a total of 22,287 adverse reaction events related to semaglutide were identified. After excluding cases with missing records and administration routes that were not relevant to the study. A total of 16,346 adverse reaction events associated with subcutaneous injection and 2,496 events associated with oral administration, which were required for this research, were retained. A detailed summary of the clinical characteristics is presented in Table 1. In the two administration routes, oral administration compared to subcutaneous injection, showed an increased proportion of adverse reaction events in males. While simultaneously reducing the proportion of adverse reaction events in females. However, irrespective of the administration route, females exhibited a higher proportion of adverse reaction events compared to males. For subcutaneous injection, the proportion in females was 63.1% compared to 32.7% in males. Similarly, for oral administration, the proportion in females was 56.5% compared to 40.1% in males. Oral administration reduced the occurrence of adverse events in middle-aged patients (18–64.9 years). However, it increased the rate of adverse events in elderly patients (>65 years). However, in both administration routes, middle-aged patients (18–64.9 years) were more prone to experiencing adverse events compared to elderly patients (>65 years). In both administration routes, the United States submitted the highest number of adverse reaction reports. In terms of adverse reaction outcomes, oral administration significantly increased the rate of hospitalization among patients. Additionally, it elevated the occurrence rate of serious adverse events (disability, life-threatening situations, and death).

**TABLE 1:** Clinical characteristics of patients with adverse events under different administration routes of Semaglutide

Characteristics	Subcutaneous of semaglutide	Oral of semagluti
X	Overall	Overall
	(N = 16,346)	(N = 2,496)
SEX,n (%)		
F	10,311 (63.1%)	1,409 (56.5%)
M	5,342 (32.7%)	1,002 (40.1%)
Missing	693 (4.2%)	85 (3.4%)
WT (kg),n (%)		
<50	11 (0.1%)	18 (0.7%)
>100	635 (3.9%)	93 (3.7%)
50–100	1,055 (6.5%)	238 (9.5%)
Missing	14,645 (89.6%)	2,147 (86.0%)
AGE (year),n (%)		
<18	11 (0.1%)	1 (0.0%)
>85	57 (0.3%)	36 (1.4%)
18–64.9	4,615 (28.2%)	650 (26.0%)
65–85	3,395 (20.8%)	556 (22.3%)
Missing	8,268 (50.6%)	1,253 (50.2%)
COUNTRY,n (%)		
US	15,286 (93.5%)	2045 (81.9%)
CN	66 (0.4%)	1 (0.0%)
CA	185 (1.1%)	5 (0.2%)
GB	82 (0.5%)	27 (1.1%)
DK	102 (0.6%)	21 (0.8%)
JP	111 (0.7%)	262 (10.5%)
Reporting year,n (%)		
2023	5,841 (36%)	729 (29%)
2022	3,954 (24%)	707 (28%)
2021	2,485 (15%)	646 (26%)
2020	2019 (12%)	412 (17%)
2019	1,037 (6%)	2 (0%)
2018	1,010 (6%)	
Outcome,n (%)		
HO	1778 (10.5%)	390 (14.8%)
DE	108 (0.6%)	43 (1.6%)
DS	191 (1.1%)	39 (1.5%)
LT	124 (0.7%)	29 (1.1%)
CA	2 (0.0%)	1 (0.0%)
RI	67 (0.4%)	10 (0.4%)
OT	3,432 (20.3%)	577 (21.9%)
Missing	11,245 (66.4%)	1,546 (58.7%)

Note: n, number of cases with available.

**FIGURE 2:**
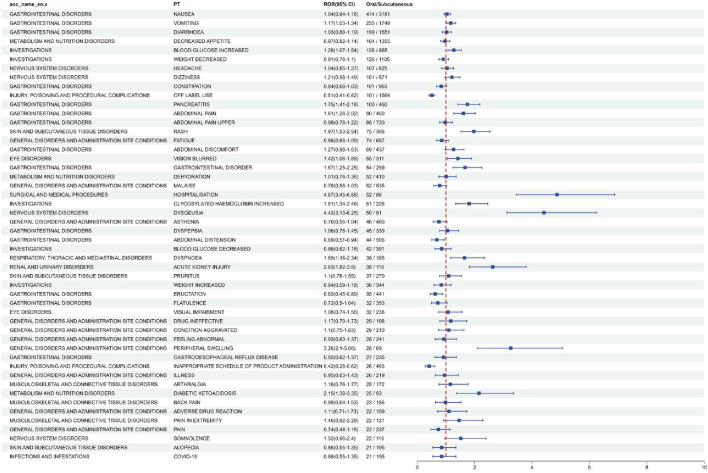
Analysis of differential risk signals for different administration routes of Semaglutide. Odds ratios (ROR) for the top 50 AEs are reported in the figure, 95% CI. PT, preferred term; SOC, system organ classes; AEs, Adverse Events.

In order to analyze the effect of different routes of administration on the adverse events of Semiglutide, we used ROR (Reporting Odds Ratio is one of the algorithms used in disproportionation analysis) to identify the adverse events under oral administration and subcutaneous injection, and ranked them according to ROR (95%CI). The top 50 (AEs) were selected and classified by system organ classes (SOC). The result is shown in Figure 2. The results of all data and the formulas for calculating the reported Odds ratio (ROR) are shown in Supplementary Table S1. From Figure 2, it can be observed that semaglutide exhibits different preferred terms (PT) and system organ classes (SOC) for adverse reactions under the two different administration routes. In comparison to subcutaneous injection, for oral administration, we consider a positive signal when the odds ratio (OR) is greater than 1 and the confidence interval does not include 1. The following adverse events, namely, vomiting, increased blood glucose, pancreatitis, abdominal pain, rash, blurred vision, gastrointestinal disorders, hospitalization, increased glycated hemoglobin, taste disturbances, respiratory difficulties, acute renal injury, peripheral edema, and diabetic ketoacidosis, are more commonly observed in oral administration. Similarly, in comparison to oral administration, for subcutaneous injection, we consider a positive signal when the odds ratio (OR) is less than 1 and the confidence interval does not include 1. The following adverse events, use methods other than those described on the label, abdominal distension, belching, and improper product handling time, are more commonly observed in subcutaneous injection.

### Comparison of AE signal intensity under different administration routes

For the data obtained from adverse event (AE) screening in different administration routes, the Reporting Odds Ratio (ROR) was employed for selection and analysis. We used the following criteria to identify positive signals: a Reporting Odds Ratio (ROR) greater than or equal to 3, with the lower limit of the 95% confidence interval (CI) being greater than 1. We then ranked the positive signals in descending order according to 95% CI and selected the top 15 AEs under each route of administration. Finally, the collected data are organized into Table 2. From Table 2, it can be observed that there are differences in the propensity to induce adverse events (AE) among different administration routes. Under oral administration, the prominently ranked adverse events (AE) include obstructive pancreatitis, thyroid cyst, neck mass, pancreatitis, gallstones, diabetic retinopathy, and gastric emptying disorders. These adverse reactions are all accompanied by warnings in the product labeling. Additionally, we have identified eight new adverse events (AE) which include ketosis, euglycemic diabetic ketoacidosis, pancreatic cancer, sixth cranial nerve palsy, hyperglycemic hyperosmolar nonketotic syndrome, elevated glycated hemoglobin, positional dizziness, and belching. Under subcutaneous injection, the prominently ranked adverse events (AE) include medullary thyroid carcinoma, gastric hypomotility, obstructive pancreatitis, diabetic retinopathy, decreased glycated hemoglobin, thyroid nodules, bile acid malabsorption, projectile vomiting, retinopathy, and neck mass. These adverse reactions are also explicitly warned about in the product labeling. Additionally, we have discovered five new adverse events (AE) which include abnormal sensation of pain, starvation ketosis acidosis, belching, follicular thyroid carcinoma, and mesenteric panniculitis. Comparative analysis revealed that oral administration is more likely to induce gastrointestinal disorders such as pancreatitis, pancreatic cancer, belching, gastric emptying disorders, and gallstones when compared to subcutaneous injection. Furthermore, oral administration is more prone to experiencing adverse events (AE) that are not listed in the product labeling. For instance, adverse events (AE) such as sixth cranial nerve palsy, hyperglycemic hyperosmolar nonketotic syndrome, elevated glycated hemoglobin, and positional dizziness are more likely to occur, which are not specifically mentioned in the product labeling. In contrast, subcutaneous injection is more likely to result in benign, malignant, and indeterminate tumors (including cystic and polypoid forms) of the thyroid such as medullary thyroid carcinoma, thyroid nodules, follicular thyroid carcinoma, and neck masses. It is also associated with endocrine system disorders.

**TABLE 2:** Comparison of AE signal intensity under different administration routes.

	PT	a	ROR	ROR (95%Cl)
	OBSTRUCTIVE PANCREATITIS	3	62.37	62.37 (20.03–194.22)
	KETOSIS	5	60.44	60.44 (25.07–145.7)
	THYROID CYST	3	48.38	48.38 (15.55–150.52)
	EUGLYCAEMIC DIABETIC KETOACIDOSIS	9	29.74	29.74 (15.45–57.26)
	ADENOCARCINOMA PANCREAS	4	29.42	29.42 (11.02–78.56)
	VITH NERVE PARALYSIS	3	26.38	26.38 (8.49–81.97)
	HYPERGLYCAEMIC HYPEROSMOLAR NONKETOTIC SYNDROME	3	25.73	25.73 (8.28–79.93)
Oral	NECK MASS	7	25.24	25.24 (12.02–53.03)
	ERUCTATION	35	21.68	21.68 (15.54–30.24)
	GLYCOSYLATED HAEMOGLOBIN INCREASED	51	21.52	21.52 (16.33–28.36)
	IMPAIRED GASTRIC EMPTYING	15	19.86	19.86 (11.96–32.99)
	PANCREATITIS	100	19.45	19.45 (15.96–23.7)
	BILE DUCT STONE	6	19.02	19.02 (8.53–42.39)
	DIABETIC RETINOPATHY	6	17.91	17.91 (8.04–39.91)
	VERTIGO POSITIONAL	3	17.41	17.41 (5.61–54.04)
	MEDULLARY THYROID CANCER	16	76.64	76.64 (46.15–127.27)
	ALLODYNIA	30	51.42	51.42 (35.64–74.17)
	STARVATION KETOACIDOSIS	3	46.85	46.85 (14.74–148.84)
	GASTRIC HYPOMOTILITY	5	36.65	36.65 (15.03–89.37)
	ERUCTATION	441	35.27	35.27 (32.06–38.79)
	OBSTRUCTIVE PANCREATITIS	12	31.85	31.85 (17.94–56.56)
	FOLLICULAR THYROID CANCER	3	24.49	24.49 (7.8–76.91)
Subcutaneous	DIABETIC RETINOPATHY	63	23.97	23.97 (18.67–30.78)
	MESENTERIC PANNICULITIS	5	23.32	23.32 (9.62–56.57)
	GLYCOSYLATED HAEMOGLOBIN DECREASED	26	22.73	22.73 (15.41–33.52)
	THYROID MASS	54	19.88	19.88 (15.18–26.02)
	BILE ACID MALABSORPTION	3	19.13	19.13 (6.11–59.9)
	VOMITING PROJECTILE	36	18.14	18.14 (13.05–25.22)
	RETINOPATHY	49	16.42	16.42 (12.38–21.78)
	NECK MASS	33	15.02	15.02 (10.65–21.19)

Note: a, number of cases with available.

### Induction time of related adverse reactions under different routes of administration

**FIGURE 3:**
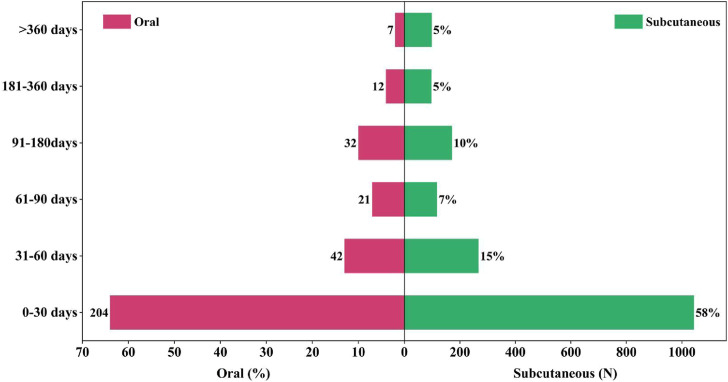
Induction time of related adverse reactions under different routes of administration.

By retrieving and summarizing the occurrence timelines of adverse reactions for semaglutide across different administration routes from the FDA Adverse Event Reporting System (FAERS) database, we can generate Figure 3. Comparative analysis reveals that oral administration, as compared to subcutaneous injection, exhibits a significant increase in the occurrence rate of adverse reactions for semaglutide within the first month (rising from 58% to 64%). Regardless of the administration route, the majority of adverse events (AE) for semaglutide occur within the first month of use. This holds true for both oral administration (64%) and subcutaneous injection (58%) of semaglutide. Under oral administration, the occurrence rate of adverse events (AE) significantly decreases in patients with a medication duration exceeding 360 days compared to subcutaneous injection (reducing from 5% to 2%). During the remaining duration of medication beyond the first month, there is little noticeable difference in the occurrence probability of adverse events between the two administration routes. However, overall, both oral administration and subcutaneous injection show a gradual decrease in the occurrence rate of adverse events over time. This may be attributed to patients gradually adapting to the medication during the treatment process or other factors.

### Time-to-onset analysis

The onset time and WSP (weighted signal proportion) analysis results for the clinical priority signals of adverse events (AE) in subcutaneous injection and oral administration are presented in Table 3. The median onset time for signals of semaglutide under different administration routes is 22 days (interquartile range [IQR]: 6–67) for subcutaneous injection and 15 days (IQR: 4–54.25) for oral administration. It is worth noting that in the WSP (weighted signal proportion) analysis evaluation, all shape parameters β and their 95% confidence interval (CI) upper limits are <1 for both administration routes. This suggests that the clinical priority signals under these two administration routes exhibit an early decline pattern.

**TABLE 3:** Time-to-onset analysis for signals with Subcutaneous and Oral prioritization.

Prioritization				Weibull distribution				Failure type
	Case	TTO (days)		Scale parameter		Shape parameter		
	n	Median (IQR)	Min–max	α	95% CI	β	95% CI	
Subcutaneous	1793	22 (6–67)	1–1789	46.4	42.50–50.30	0.58	0.56–0.60	Early failure
Oral	318	15 (4–54.25)	1–1,006	33.36	27.08–39.63	0.61	0.57–0.67	Early failure

Note: n, number of cases with available time-to-onset; IQR, interquartile range; TTO, Time-to-onset.

In order to ascertain potential differences in the reporting rates of Simeglutide based on patient gender, age, indications, and treatment duration. We conducted subgroup analyses based on patient gender (male and female), age groups (<18 years, 18–65 years, and >65 years), as well as two indications (obesity and diabetes) and the duration of drug treatment. The results of these subgroup analyses are presented in Table 4, Table 5, Table 6, and Table 7, respectively, illustrating the stratification based on patient gender, age, indications, and treatment duration. Differences were observed in the occurrence and reporting rates of adverse events related to the reporting odds ratio (ROR) between males and females. Particularly noteworthy is the occurrence of penile inflammation as an adverse event in males, which was not explicitly warned in the product labeling but exhibited a relatively higher probability of occurrence. In terms of age, compared to elderly patients, the incidence of adverse events is generally higher among middle-aged and young adults. Particularly notable is the increased occurrence rate among this age group in conditions such as starvation ketosis acidosis, sensory abnormalities, and medullary thyroid carcinoma. In different indications, the incidence of medullary thyroid carcinoma is twice as high in diabetes patients compared to obesity patients. Additionally, diabetes patients are more prone to experiencing blood glucose fluctuations and diabetes-related complications as adverse events. Overall, the incidence of adverse events is generally higher in diabetes patients compared to obesity patients. In terms of treatment duration, the incidence of adverse events within a treatment cycle (typically 4 weeks) is significantly lower compared to other time periods. Moreover, the severity of adverse events occurring within a treatment cycle is also relatively mild compared to other time periods. This suggests that patients gradually adapt to the medication when they start the treatment, resulting in a lower incidence and severity of adverse events.

**TABLE 4:** Sex-reported Odds ratio (ROR) for commonly reported adverse events with Semaglutide

	PT	a	ROR	ROR (95%Cl)
	MEDULLARY THYROID CANCER	14	77.24	77.24 (45.02–132.51)
	STARVATION KETOACIDOSIS	4	73.65	73.65 (26.87–201.9)
	ALLODYNIA	27	53.52	53.52 (36.4–78.67)
	OBSTRUCTIVE PANCREATITIS	14	43.4	43.4 (25.47–73.94)
	FOOD AVERSION	35	36.73	36.73 (26.24–51.41)
	POSTPRANDIAL HYPOGLYCAEMIA	3	30.79	30.79 (9.79–96.79)
	ERUCTATION	332	30.57	30.57 (27.4–34.1)
Female	GASTRIC HYPOMOTILITY	3	25.21	25.21 (8.04–79.05)
	THYROID MASS	54	23.1	23.1 (17.65–30.24)
	DUODENOGASTRIC REFLUX	3	23.04	23.04 (7.35–72.19)
	BILE ACID MALABSORPTION	3	22.22	22.22 (7.1–69.61)
	MESENTERIC PANNICULITIS	4	21.59	21.59 (8.03–58)
	VOMITING PROJECTILE	35	20.48	20.48 (14.66–28.61)
	GLYCOSYLATED HAEMOGLOBIN DECREASED	19	19.19	19.19 (12.2–30.19)
	PAPILLARY THYROID CANCER	21	18.9	18.9 (12.28–29.08)
	PENILE DERMATITIS	4	132.89	132.89 (48.58–363.48)
	MEDULLARY THYROID CANCER	9	95.39	95.39 (49.01–185.68)
	MESENTERIC PANNICULITIS	6	64.14	64.14 (28.51–144.27)
	OBSTRUCTIVE PANCREATITIS	9	54.11	54.11 (27.95–104.76)
	GASTRIC HYPOMOTILITY	3	49.5	49.5 (15.78–155.23)
	ALLODYNIA	13	49.49	49.49 (28.58–85.72)
	ERUCTATION	204	36.6	36.6 (31.85–42.05)
Male	FOOD AVERSION	14	28.34	28.34 (16.73–48)
	DIABETIC RETINOPATHY	30	25.75	25.75 (17.96–36.9)
	NECK MASS	24	24.84	24.84 (16.62–37.15)
	GLYCOSYLATED HAEMOGLOBIN ABNORMAL	8	24.41	24.41 (12.16–48.98)
	HICCUPS	61	22.6	22.6 (17.55–29.08)
	RETINOPATHY	28	21.27	21.27 (14.66–30.87)
	FLOPPY IRIS SYNDROME	3	21.07	21.07 (6.76–65.66)
	PANCREATITIS NECROTISING	16	20	20 (12.22–32.71)

Note: a, number of cases with available; PT, preferred term.

**TABLE 5:** Age-reported Odds ratio (ROR) for commonly reported adverse events with Semaglutide

AGE (years)	PT	a	ROR	ROR (95%Cl)
	NAUSEA	4	7.19	7.19 (2.58–20.04)
<18	DIARRHOEA	3	6.61	6.61 (2.05–21.29)
	STARVATION KETOACIDOSIS	5	165.17	165.17 (66.57–409.82)
	ALLODYNIA	31	109.37	109.37 (76.24–156.9)
	MEDULLARY THYROID CANCER	11	105.88	105.88 (57.81–193.9)
	MESENTERIC PANNICULITIS	8	77.67	77.67 (38.37–157.21)
	OBSTRUCTIVE PANCREATITIS	10	54.26	54.26 (28.97–101.62)
	GASTRIC HYPOMOTILITY	3	44.56	44.56 (14.21–139.74)
	FOOD AVERSION	21	38.5	38.5 (25.01–59.28)
18–65	ERUCTATION	231	37.39	37.39 (32.81–42.61)
	VAGUS NERVE DISORDER	3	35.5	35.5 (11.35–111.09)
	PANCREATIC INJURY	3	29.91	29.91 (9.57–93.44)
	VOMITING PROJECTILE	27	27.83	27.83 (19.04–40.69)
	IMPAIRED GASTRIC EMPTYING	72	24.7	24.7 (19.57–31.17)
	PAPILLARY THYROID CANCER	15	23.76	23.76 (14.28–39.52)
	EUGLYCAEMIC DIABETIC KETOACIDOSIS	25	21.32	21.32 (14.38–31.62)
	THYROID MASS	25	18.71	18.71 (12.62–27.75)
	OBSTRUCTIVE PANCREATITIS	7	58.39	58.39 (27.66–123.28)
	FOOD AVERSION	15	42.38	42.38 (25.47–70.54)
	FLOPPY IRIS SYNDROME	4	39.29	39.29 (14.66–105.28)
	ERUCTATION	139	34.59	34.59 (29.24–40.91)
	ADENOCARCINOMA PANCREAS	9	26.42	26.42 (13.71–50.92)
	ALLODYNIA	5	26.21	26.21 (10.87–63.19)
	GLYCOSYLATED HAEMOGLOBIN ABNORMAL	5	21.19	21.19 (8.79–51.05)
>65	KETOSIS	4	19.18	19.18 (7.18–51.24)
	NECK MASS	12	17.23	17.23 (9.77–30.38)
	BENIGN NEOPLASM OF THYROID GLAND	3	15.92	15.92 (5.12–49.49)
	PANCREATIC MASS	4	15.58	15.58 (5.83–41.61)
	DIABETIC RETINOPATHY	13	15.45	15.45 (8.96–26.65)
	VOMITING PROJECTILE	9	14.24	14.24 (7.4–27.41)
	NEOVASCULAR AGE-RELATED MACULAR DEGENERATION	4	13.64	13.64 (5.11–36.43)
	PANCREATIC FAILURE	5	13.57	13.57 (5.64–32.67)

Note: a, number of cases with available; PT, preferred term.

**TABLE 6:** Indications Reporting Odds Ratio (ROR) for commonly reported adverse events with Semaglutide

	PT	a	ROR	ROR (95%Cl)
	MEDULLARY THYROID CANCER	11	123.97	123.97 (67.69–227.05)
	ALLODYNIA	11	44	44 (24.24–79.87)
	ERUCTATION	200	37.82	37.82 (32.87–43.52)
	PANCREATIC INJURY	3	35.02	35.02 (11.21–109.41)
	OBSTRUCTIVE PANCREATITIS	4	25.04	25.04 (9.35–67.03)
	DIABETIC RETINOPATHY	26	23.48	23.48 (15.96–34.56)
	THYROID MASS	23	20.15	20.15 (13.36–30.37)
DIABETES MELLITUS	RETINOPATHY	23	18.39	18.39 (12.2–27.72)
	GLYCOSYLATED HAEMOGLOBIN DECREASED	8	16.57	16.57 (8.27–33.22)
	VOMITING PROJECTILE	13	15.58	15.58 (9.03–26.88)
	HICCUPS	37	14.38	14.38 (10.41–19.87)
	IMPAIRED GASTRIC EMPTYING	34	13.56	13.56 (9.68–18.99)
	PANCREATITIS NECROTISING	10	13.13	13.13 (7.05–24.45)
	GLYCOSYLATED HAEMOGLOBIN ABNORMAL	4	12.8	12.8 (4.79–34.18)
	GLYCOSYLATED HAEMOGLOBIN INCREASED	101	12.79	12.79 (10.52–15.56)
	STARVATION KETOACIDOSIS	4	323.69	323.69 (118.06–887.5)
	ALLODYNIA	24	208.42	208.42 (138.58–313.44)
	MEDULLARY THYROID CANCER	3	69.36	69.36 (22.21–216.62)
	OBSTRUCTIVE PANCREATITIS	4	53.17	53.17 (19.86–142.36)
	FOOD AVERSION	10	45.17	45.17 (24.23–84.19)
	FOOD CRAVING	20	34.33	34.33 (22.11–53.31)
	MYOGLOBINURIA	3	33.76	33.76 (10.85–105.05)
OBESITY	BINGE EATING	5	33.52	33.52 (13.91–80.76)
	ERUCTATION	68	26.97	26.97 (21.23–34.25)
	VOMITING PROJECTILE	10	25.42	25.42 (13.65–47.33)
	BILIARY COLIC	13	24.74	24.74 (14.34–42.68)
	SENSITIVE SKIN	19	20.95	20.95 (13.35–32.89)
	PAPILLARY THYROID CANCER	5	19.54	19.54 (8.12–47.04)
	IMPAIRED GASTRIC EMPTYING	22	18.6	18.6 (12.23–28.28)
	SKIN SENSITISATION	3	18.22	18.22 (5.86–56.61)

Note: a, number of cases with available; PT, preferred term.

**TABLE 7:** Time-of-treatment Reporting Odds Ratio (ROR) for commonly reported adverse events with Semaglutide

During	PT	a	ROR	ROR (95%Cl)
	VOMITING PROJECTILE	11	54.44	54.44 (30.09–98.52)
	KETOSIS	3	44.82	44.82 (14.42–139.36)
	ERUCTATION	37	28.47	28.47 (20.59–39.36)
	FOOD AVERSION	3	26.18	26.18 (8.43–81.31)
	VITREOUS HAEMORRHAGE	5	22.71	22.71 (9.44–54.64)
	EUGLYCAEMIC DIABETIC KETOACIDOSIS	5	20.45	20.45 (8.5–49.19)
	DIABETIC RETINOPATHY	5	18.5	18.5 (7.69–44.49)
4 weeks	VITREOUS DETACHMENT	3	17.77	17.77 (5.72–55.17)
	PHARYNGEAL SWELLING	9	14.45	14.45 (7.51–27.81)
	DIABETIC KETOACIDOSIS	27	14.22	14.22 (9.74–20.76)
	NECK MASS	3	13.38	13.38 (4.31–41.54)
	IMPAIRED GASTRIC EMPTYING	8	13.11	13.11 (6.55–26.24)
	ILEUS PARALYTIC	4	11.47	11.47 (4.3–30.59)
	HYPERAESTHESIA	8	11.43	11.43 (5.71–22.87)
	PANCREATIC DISORDER	4	10.92	10.92 (4.1–29.12)
	MEDULLARY THYROID CANCER	6	410.24	410.24 (182.31–923.15)
	OBSTRUCTIVE PANCREATITIS	4	155.15	155.15 (57.92–415.59)
	ALLODYNIA	4	98.06	98.06 (36.67–262.22)
	FOOD AVERSION	5	65.61	65.61 (27.24–158.03)
	PANCREATITIS CHRONIC	7	57.28	57.28 (27.25–120.41)
	PANCREATIC CARCINOMA METASTATIC	8	50.1	50.1 (25.01–100.38)
	PAPILLARY THYROID CANCER	4	45.59	45.59 (17.07–121.7)
>4 weeks	MACULAR HOLE	3	42.43	42.43 (13.66–131.82)
	CHOLECYSTITIS INFECTIVE	6	41.88	41.88 (18.78–93.38)
	ADENOCARCINOMA PANCREAS	3	41.02	41.02 (13.2–127.44)
	BILIARY COLIC	7	38.79	38.79 (18.46–81.51)
	IMPAIRED GASTRIC EMPTYING	14	34.54	34.54 (20.42–58.42)
	DIABETIC RETINOPATHY	6	33.33	33.33 (14.95–74.31)
	THYROID MASS	6	32.37	32.37 (14.52–72.17)
	LIPASE INCREASED	11	26.81	26.81 (14.83–48.49)

Note: a, number of cases with available; PT, preferred term.

## Discussion

This study conducted a comparative analysis of adverse event occurrences for semaglutide under different administration routes by analyzing the data from the United States Food and Drug Administration’s (FDA) Adverse Event Reporting System (FAERS). The aim was to provide an overview of adverse reaction events associated with semaglutide. The results of various comparative analyses indicate differences in the occurrence rate, types, and timing of adverse events under different administration routes. Through demographic analysis, we discovered that oral administration is more likely to lead to adverse events in males and reduce the risk of adverse events in females. However, regardless of whether it is oral administration or subcutaneous injection, the proportion of adverse events in females is higher than that in males. This suggests that females may be more sensitive to semaglutide. It is important to note that in the current middle-aged population, there is a significant proportion of individuals with type 2 diabetes and obesity. Compared to subcutaneous injection, oral administration significantly reduces the risk of adverse events in middle-aged patients (18–64.9 years old). However, it increases the occurrence rate of adverse events in elderly patients (65 years and older). However, it is important to note that both middle-aged and elderly patients are more prone to adverse events, regardless of whether they receive oral or subcutaneous administration. Subgroup analysis revealed that subcutaneous injection of semaglutide is more likely to cause adverse events related to the endocrine system compared to oral administration. According to the data from the FDA Adverse Event Reporting System, oral administration of semaglutide has fewer hidden risks associated with the endocrine system compared to subcutaneous injection. This indicates that the choice of administration route has a significant impact on whether patients experience endocrine-related issues. Furthermore, oral administration significantly accelerates the onset time of adverse reactions compared to subcutaneous injection. This is because oral administration allows the drug to enter the circulatory system more rapidly, thereby exerting its effects more quickly. Therefore, when using semaglutide, adverse reactions occur earlier with oral administration compared to subcutaneous injection (Merck, 2017).

Our study findings confirm the conclusions drawn from current epidemiological research. These findings align with previous studies and provide further support for the impact of different administration routes on the occurrence of adverse events. For example, studies have shown a significant association between oral administration of semaglutide and gastrointestinal adverse events (Shu et al., 2022b; Liu et al., 2022). Oral administration of semaglutide has been found to be significantly associated with gastrointestinal system disorders such as pancreatitis, pancreatic cancer, eructation, gastric emptying disorder, and gallstones. Studies have indicated a possible association between long-term use of GLP-1 receptor agonists (GLP-1RAs) and an increased risk of pancreatitis, although the underlying mechanism remains unclear (Gier et al., 2012a; Andreadis et al., 2018a). This may be attributed to the inhibitory effects of semaglutide on gastric emptying and slowing of gastrointestinal motility, resulting in prolonged food transit time and stimulation of gastrointestinal sensory organs (Nachnani et al., 2010; Andreadis et al., 2018b; Zhou et al., 2022). Subcutaneous injection of semaglutide has been associated with an increased risk of benign and malignant tumors (including cystic and polypoid forms) and endocrine system disorders such as medullary thyroid carcinoma, thyroid nodules, thyroid follicular carcinoma, neck masses, and tumors of uncertain nature. However, current scientific research has not definitively established a direct causal relationship between subcutaneous injection of semaglutide and endocrine system disorders such as medullary thyroid carcinoma (MTC) (Carlomagno et al., 1995; Freichel et al., 1996; Ezzat et al., 2005). Nevertheless, certain findings from animal experiments and clinical studies involving semaglutide have raised concerns regarding this issue. In animal experiments, some studies have shown that administration of high doses of GLP-1 receptor agonists, such as semaglutide, resulted in thyroid tissue hyperplasia and increased C-cell population in mice. C-cells are responsible for producing calcitonin in the thyroid gland, and MTC is precisely characterized by malignant proliferation of C-cells. These experimental findings have raised concerns regarding a potential association between GLP-1 receptor agonists and C-cell hyperplasia and MTC (DrostenM et al., 2004; Gallel et al., 2008; Li et al., 2023). In clinical research, some case reports have mentioned rare instances of MTC in patients receiving long-term treatment with GLP-1 receptor agonists, including semaglutide. These reports do not establish a direct causative link between semaglutide and MTC, but they have raised concerns regarding the thyroid safety of patients using this class of medications (Machens et al., 2009; Bjerre Knudsen et al., 2010; Yang et al., 2022).

Our study also assessed the potential differences in adverse event reporting based on patient gender, age, indications, and treatment duration. We found variations in adverse events across different genders, age groups, indications, and treatment durations. The occurrence rates of the same adverse event vary significantly among different genders, age groups, and treatment indications. In terms of treatment duration, the incidence of adverse events within a treatment cycle (typically 4 weeks) is significantly lower compared to other time periods. Moreover, the severity of adverse events occurring within a treatment cycle is also relatively mild compared to other time periods. The observed disparities serve as valuable warnings for populations at potential risk, enabling physicians to provide more tailored medication recommendations for different demographic groups and ensure continuous monitoring and management of the risk of adverse events throughout the entire treatment process.

### Limitations

Indeed, while mining and studying real-world data based on the FDA Adverse Event Reporting System (FAERS) database have strategic advantages, it is important to acknowledge that all drug safety databases have inherent limitations. Firstly, the system may be subject to limitations due to voluntary reporting, thereby posing risks of reporting bias and underreporting. Consequently, obtaining comprehensive and accurate data on adverse events may be challenging. Secondly, due to the nature of pharmacovigilance analysis that solely provides statistical associations, adverse event reporting system data cannot offer direct evidence of causality. While this system can identify adverse events associated with drug use, it cannot prove that these events are caused solely by the medication itself, nor can it exclude the potential influence of other underlying factors or concomitant drug use on the occurrence of adverse events. Furthermore, it is important to note that research findings from the FDA Adverse Event Reporting System may have certain limitations and may not be easily generalizable to the entire population. The data from the reporting system often reflect specific patient populations or specific time periods. Therefore, before extrapolating the results to a broader population, further validation and research are necessary. Moreover, our focus was solely on the impact of different routes of administration on adverse events (AEs), without excluding other confounding factors such as adherence to prescribed administration schedules or dosages, which could potentially influence (AEs). Therefore, further experimental exploration, clinical trials, case-control studies, and cohort studies are needed to validate the observed correlations.

### Clinical significance and pharmacovigilance

Our pharmacovigilance study provides an analysis based on a large sample of real-world safety data, investigating the occurrence of different adverse events associated with the use of semaglutide via various routes of administration. We also considered different genders and populations, and observed variations in the proportion of adverse events occurring with different modes of administration. Adverse events can vary depending on the route of administration. Oral administration is more likely to result in gastrointestinal adverse events, while subcutaneous injection is more likely to lead to endocrine system-related adverse events. Compared to subcutaneous injection, oral administration represents a novel option that can enhance patient acceptance and adherence. It has the potential to facilitate broader adoption of semaglutide in clinical practice. The disproportionation signals under both routes were characterized by early failure types, suggesting that most patients experienced adverse events within 1 month of oral or subcutaneous semaglutide treatment, and that the risk of adverse events gradually decreased over time. Our findings are important for clinical practice and pharmacovigilance to fully evaluate adverse events that occur under different administration routes of semaglutide. Clinicians and patients should consider the risk of adverse events and clinical benefits when choosing the route of administration.

The administration of Simeglutide through various routes may potentially elicit distinct adverse reactions, which could have implications for the safety of both physicians and patients. For physicians, it is imperative to ensure proper handling of injection devices, particularly when administering subcutaneous dosages, as strict adherence to aseptic techniques is essential to minimize the risk of infections. Moreover, physicians must acquire a comprehensive understanding of the distinctive characteristics of adverse reactions that may arise from Simeglutide administration via different routes. This knowledge enables them to promptly identify and manage any potential adverse reactions that patients may experience across various administration methods, thereby safeguarding patient safety. For patients, it is crucial to adhere to the physician’s instructions accurately when it comes to medication administration via different routes, ensuring the correct dosage and timing are followed diligently. Simultaneously, patients should also acquire knowledge about the gastrointestinal adverse reactions that may arise from oral administration and the endocrine system adverse events that may occur from subcutaneous injections. This understanding enables them to closely monitor their own bodily responses, promptly detect, and address any potential adverse reactions that may arise. In the event of severe adverse reactions, patients should promptly inform their healthcare provider or seek medical assistance to ensure their personal safety. Especially during subcutaneous injections, it is essential to consider the assistance of a physician or the training of family members in the correct injection techniques and skills for elderly patients with limited mobility, paralysis, or bedridden conditions. This precautionary measure aims to minimize the risk of injection errors, reduce the potential for infections, and mitigate the occurrence of adverse reactions. By implementing such measures, the accuracy and safety of injections can be ensured, thereby protecting patients from the adverse effects that may arise.

In summary, healthcare professionals and patients need to collaborate mutually to ensure the proper administration process and monitoring, thereby reducing the risk of adverse reactions. Physicians should provide detailed medication information tailored to the individual patient’s condition and offer guidance on the use of different administration routes. Patients, on their part, should actively cooperate with the physician’s treatment plan, follow the prescribed instructions, and promptly report any discomfort or abnormal reactions to their healthcare provider. Through active communication and collaboration between physicians and patients, the safety of Simeglutide treatment across various administration routes can be maximized.

## Conclusion

In summary, as Semaglutide has been widely used due to its significant hypoglycemic effect and weight loss efficacy, clinicians should fully understand the potential adverse events that may be caused by different drug dosage forms when selecting drug dosage forms and routes of administration. Provide personalized recommendations to patients based on their condition, individual differences, and drug characteristics to reduce the risk of adverse events and maximize efficacy.

## Data Availability

The datasets analyzed during the current study are included in the article/Supplementary Material, further inquiries can be directed to the corresponding.

## Abbreviations

GLP-1RA:glucagon-like peptide-1 receptor agonist,FDA:the adverse event reporting system of the U.S. Food and Drug, ROR:, reporting odds ratio, AE:signals of adverse events, PT:preferred terms, SOC:system organ classes, OR: odds ratio, MTC:medullary thyroid carcinoma.
